# Genomic and microbial factors affect the prognosis of anti-pd-1 immunotherapy in nasopharyngeal carcinoma

**DOI:** 10.3389/fonc.2022.953884

**Published:** 2022-08-17

**Authors:** Liqin Xu, Yuxiang Ma, Chao Fang, Zhuobing Peng, Fangfang Gao, Janne Marie Moll, Shishang Qin, Qichao Yu, Yong Hou, Karsten Kristiansen, Wenfeng Fang, Susanne Brix, Li Zhang

**Affiliations:** ^1^ BGI-Shenzhen, Shenzhen, China; ^2^ Department of Biotechnology and Biomedicine, Technical University of Denmark, Kgs. Lyngby, Denmark; ^3^ Latvia MGI Tech SIA, Marupe, Latvia; ^4^ State Key Laboratory of Oncology in South China, Collaborative Innovation Center of Cancer Medicine, Sun Yat-sen University Cancer Center, Guangzhou, China; ^5^ China National GeneBank, BGI-Shenzhen, Shenzhen, China; ^6^ Laboratory of Genomics and Molecular Biomedicine, Department of Biology, University of Copenhagen, Copenhagen, Denmark; ^7^ College of Life Sciences, University of Chinese Academy of Sciences, Beijing, China; ^8^ Institute of Metagenomics, Qingdao-Europe Advanced Institute for Life Sciences, Qingdao, China

**Keywords:** NPC, immunotherapy, PD-1, TMB, HLA, EBV, gut microbiota

## Abstract

Antibodies targeting the programmed cell death protein-1 (PD-1) molecule have been reported to hold promising antitumor activities in patients with nasopharyngeal carcinoma (NPC). However, only a small subset of NPC patients benefits from the anti-PD-1 monotherapy and factors that affect the treatment response need further investigation. This study aimed to examine the impact of multiple genetic and environmental factors on outcome of anti-PD-1 immunotherapy by identifying tumor size, tumor mutation burden (TMB) based on whole exon sequencing, human leukocyte antigen class I (HLA-I) homo-/heterozygosity and supertypes, blood Epstein-Barr virus (EBV) DNA load, T cell proportions, and interferon-γ(IFN-γ) levels in a cohort of 57 NPC patients that received Nivolumab or Camrelizumab treatment. Moreover, we profiled the longitudinal changes in gut microbiota composition using shotgun metagenomics sequencing. We observed that high TMB combined with HLA-I heterozygosity was associated with improved clinical outcomes. In agreement with previous studies, we found that patients with higher plasma EBV DNA load showed worse progression-free survival. We found no evidence for an effect of gut bacterial diversity on the treatment response, but identified a higher abundance of seven specific gut bacteria at baseline of non-responders, including *Blautia wexlera* and *Blautia obeum*, as well as four other bacteria belonging to the *Clostridiales* order, and one *Erysipelatoclostridium*. Combined, this study provides insight into the influence of several genetic and environmental factors on anti-PD-1 immunotherapy responses in NPC patients.

## Introduction

Nasopharyngeal carcinoma (NPC) is globally considered a rare cancer, with an estimated age‐standardized incidence of 2.2 per 100,000 among men and 0.8 per 100,000 among women in 2020 (1). However, the incidence rates of NPC in South China are 30 per 100,000 for men and 11 per 100,000 for women (2). Migrant studies showed that Chinese born in the United States (US) retained an increased risk of NPC. Even third-generation US Chinese (parents born in the US) have a 10-fold higher risk than US white Americans (3), suggesting that genetics play a key role in NPC risk. Furthermore, NPC is an Epstein-Barr virus (EBV)-associated malignancy, and extensive evidence indicates that EBV is a potential cause of NPC (4), indicating that environmental exposures also play a role in the NPC etiology.

NPC is a highly chemosensitive cancer and gemcitabine plus cisplatin has been established as the standard first-line treatment in recurrent or metastatic NPC (RM-NPC). Still, the outcome is poor, with a median overall survival of 29 months (5). More recently, four different anti-PD-1 antibodies, Pembrolizumab, Nivolumab, Camrelizumab, Toripalimab, and Tislelizumab, have been used to treat RM-NPC and shown to significantly improve survival in a small proportion of the patients (6–11). However, the objective response rate (ORR) with these four anti-PD-1 monotherapies is low, ranging from 19.0% to 34.1% in pretreated RM-NPC patients (12). Therefore, predictive biomarkers for efficacy are imperative for RM-NPC patients receiving anti-PD-1 treatment.

Tumor mutational burden (TMB) has been broadly investigated in most human cancers and reported to be significantly correlated with anti-PD-1 therapy ORR (13–15). However, TMB is relatively low in NPC (median 3.05 mutations/Megabase (Mb)) compared with the median across all 35 major tumor types (3.48 mutations/Mb) (14). In the study of Toripalimab-treated RM-NPC patients, no correlation was found between TMB and progression-free survival (10). Still, patients with low circulating levels of EBV DNA were found to have better ORR than those with high levels of EBV DNA (10). Mutated cancer cells presenting tumor antigens by human leukocyte antigen class I (HLA-I) could be identified and killed by CD8^+^ T cells, and low heterozygosity of HLA-I has been associated with poor outcomes for melanoma patients treated with anti-PD-1 (16). Due to a low number of studies, the effect of TMB, EBV levels and HLA-I heterozygosity on the response to anti-PD-1 therapy in NPC patients warrants further study.

The gut microbiota has been reported to affect the clinical outcome of immune checkpoint inhibitors therapy in various cancer patients. A few bacterial species, including *Faecalibacterium prausnitzii (* 17) and *Bifidobacterium longum* (18), were found to be predictors of responders to anti-PD-1 treatment in melanoma patients. Another study identified *Akkermansia muciniphila* to be correlated with better clinical response to anti-PD-1 therapy in patients with non-small cell lung cancer (NSCLC) and renal cell carcinoma (RCC) (19). In addition, the gut microbiota was reported to affect the response of anti-PD-1 immunotherapy in patients with hepatocellular carcinoma (20) and gastrointestinal cancer (21). Of note, the potential for enhancing cancer immunotherapy by modulating the gut microbiota has been highlighted by two recent clinical trials applying fecal microbiota transplantation (FMT) to melanoma patients that received anti-PD-1 therapy (22, 23). Still, the association between the gut microbiota and the response to anti-PD-1 treatment in the RM-NPC patients remains unclear.

In this study of anti-PD-1 treated NPC patients, we evaluated the association between clinical outcomes and multiple genetic and environmental factors, including TMB, HLA-I, EBV, and the gut microbiota. Our findings provide information of importance for selecting the NPC patients that may benefit the most from anti-PD-1 immunotherapy.

## Materials and methods

### Patients and medications

A cohort of 57 patients was included in this study. All patients were clinically diagnosed with RM-NPC at Sun Yat-sen University Cancer Center (SYSUCC), China. Thirty-four patients received Camrelizumab (SHR-1210) monotherapy (HengRui Medicine Co., Jiangsu, China) with a dose of 200 mg every second week. Twenty-three patients received Nivolumab monotherapy (Bristol-Myers Squibb, New York. US.) with a dose of 240 mg every second week. All the patients were enrolled in clinical trials registered at ClinicalTrials.gov (NCT02721589 (Camrelizumab monotherapy phase I trial), and NCT02593786 (Nivolumab monotherapy phase I/II trial)). Response evaluation was assessed according to the Response Evaluation Criteria in Solid Tumors (RECIST) version 1.1. The overall treatment information and response results are provided in **Supplementary Table S1** and **Supplementary Figure S1A**.

### Whole exome sequencing of tumor tissue

In 46 of the 57 patients, genomic DNA from formalin-fixed paraffin-embedded (FFPE) samples were extracted using QIAamp DNA FFPE Tissue Kit (Qiagen, USA). Normal control DNA was extracted from peripheral blood of patients before therapy by using DNeasy blood & tissue kits (Qiagen, USA). Library generation and whole exome sequencing (WES) were performed as described (24). In short, genomic DNA was fragmented by M220 Focused-ultrasonicator (Covaris, UK), followed by whole-genome library preparation using HyperPrep Kit (KAPA Biosystems). Exome libraries were enriched using the Agilent V6 Kit (Agilent Inc.) and sequenced on the Illumina HiSeq 4000 platform. Suffcient numbers of paired-end 150-bp reads were generated to reach the mean coverage of ~200X for the tumor samples and ~60X for the normal blood samples.

### Whole exome sequencing data analysis and TMB profiling

WES data were processed to detect genetic variants following the BWA-PICARD/GATK-strelka pipeline. Briefly, paired-end sequencing data were aligned to the reference human genome (build hg19) using the Burrows-Wheeler Aligner (version 0.7.12). The aligned reads were sorted and de-duplicated by PICARD (http://broadinstitute.github.io/picard). GATK (version 3.7) was used to perform multiple reads alignment around putative indel and recalibrate base quality before variants detection. The analysis-ready bam files were input into strelka (25) (version 1) to call somatic single nucleotide variants (SNVs) and insertion-deletion mutations (INDELs). Four exclusion filters were applied for somatic SNV calling: (i) less than 5 alternate reads in tumor samples; (ii) less than 5% variant allele frequency (VAF); (iii) less than 15 reads in total in the tumor and control samples; (iv) presence of the variant in the 1000 Genomes project at a frequency > 1%. Annovar (version 2015-06-17) was applied for somatic mutations annotation.

Tumor mutational burden (TMB) was defined as the number of somatic, coding, base substitution, and indel mutations per megabase of genome examined, according to the method by Chalmers et al. (26). We calculated TMB by dividing the number of somatic non-synonymous mutations by the total length of sufficiently sequenced regions captured by Agilent V6 Kit (regions with > 30 sequencing depth in both tumor and matched normal sample) measured in megabase. The TMB profiles of each patient are provided in **Supplementary Table S1**.

### HLA-I profiling

We performed high-resolution HLA-I genotyping from 46 patients with peripheral blood collected before they received therapy. Extracted DNA from peripheral blood was processed with hybridization-based capture methods, and targeted HLA-A, HLA-B, and HLA-C gene sequencing was performed by next-generation sequencing as discribed (27). The HLA-I alleles were classified into twelve supertypes based on similar peptide-anchor-binding specificities (28).

### Plasma EBV DNA profiling and IFN-γ levels

Plasma EBV DNA load was quantified by real-time quantitative polymerase chain reaction (RT-qPCR; EBV-DNA Fluorescence Quantitative PCR Detection Kit, Targene, Guangzhou, China) in the Department of Molecular Diagnosis at SYSUCC. Peripheral blood (3 ml) from patients was collected into EDTA-containing tubes, plasma was isolated after centrifuging peripheral blood at 3,000 rpm for 5 min. DNA from plasma samples were then extracted using the Qiamp Blood Kit (Qiagen, Hilden, Germany) following the user manual. The copy number of target gene EBV-LP was measured by absolute quantitative PCR method. Blood IFN-γ was quantified by ELISA kits (BD Pharmingen, San Diego, CA) according to the manufacturer’s protocol.

### Lymphocyte phenotyping by flow cytometry

Anti-CD45-PerCP-Cy5.5 (BD Biosciences) was used to gate leukocytes, anti-CD3-FITC (BD Biosciences) was used for the identification of T lymphocytes, anti-CD4-PE-Cy7 (BD Biosciences) for detecting T-helper lymphocytes, anti-CD8-APC-Cy7 (BD Biosciences) for the detection of cytotoxic T lymphocytes. The cells were analyzed by a FACSCalibur flow cytometer using FACSDiva clinical software (version 9.0, BD Biosciences) following the user manual. The percentage of each T cell lymphocyte subset in human peripheral blood is provided in **Supplementary Table S2**.

### Fecal sample collection and metagenomics analysis

Feces was collected from 50 of the 57 patients, at the timepoint showed in **Supplementary Figure S2**. The relationship between fecal sample, tumor sample and HLA data collection was showed in **Supplementary Figure S1B**. Freshly collected fecal samples were immediately frozen and stored at -80°C until extraction. Total fecal DNA was extracted and sequenced using BGISEQ-500 following the standard protocol as described (29). After data quality control, the filtered gut microbiome DNA fragments were mapped to the integrated gene catalogue (IGC) 9.9M reference and quantitated as described (30). The metagenomic species (MGS) profile was generated based on a 1507 MGS catalogue clustered from 2307 human gut microbiome samples (31) and annotated using NCBI RefSeq and GTDB-Tk as described by Moll et al. (32). Taxonomical annotations were manually curated to ensure congruence between all taxonomical levels for a given MGS.

### Statistical analysis

Statistical analyses were mainly performed in the program R version 3.4.3 with the following packages: spearman correlation performed by cor(); Wilcoxon test performed by wilcox.test(); Alpha diversity was calculated by the Shannon index, while Bray-Curtis dissimilarity was used to compute the beta diversity. The method used to calculate the reporter-score was described previously (33).

## Results

### Effect of tumor mutation burden and HLA-I homozygosity

**Figure 1:**
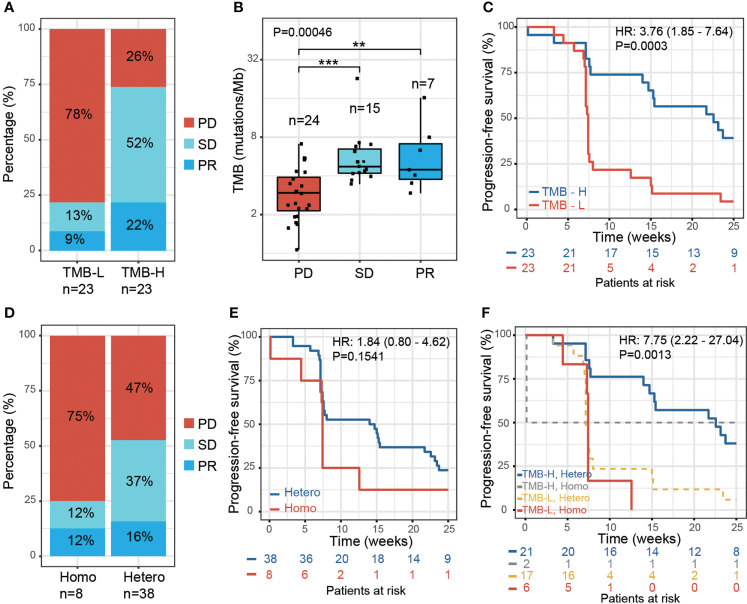
Effect of tumor mutation burden and HLA-I heterozygosity on survival after anti-PD-1 treatment. **(A)** The percentage of PR, SD and PD in patients with low TMB (TMB-L, TMB < 4, n=23) and high TMB (TMB-H, TMB > 4, n=23). **(B)** A boxplot of tumor mutation burden for PD (n=24), SD (n=15) and PR (n=7) patients, *P* values were calculated using the Kruskal-Wallis test. Asterisks denote pairwise group comparisons by Dunn’s test (**P<0.01; ***P<0.001). **(C)** Progression-free survival analysis of patients with high and low tumor mutation burden. **(D)** The percentage of PR, SD and PD in the patients with homozygosity in at least one HLA-I locus (Homo, n=8) and with heterozygosity at all HLA-I loci (Hetero, n=38). **(E)** Progression-free survival analysis of patients with homozygosity compared with heterozygosity in the HLA-I allele. **(F)** Progression-free survival analysis of patients with high tumor mutation burden and heterozygosity at all HLA-I loci (TMB-H, Hetero, n=21) compared with patients that have low tumor mutation burden and are homozygous for at least one HLA-I locus (TMB-L, Homo, n=6).

To study the influence of genetic factors on therapy efficacy in NPC patients, we first examined the association between TMB and the response to therapy. We performed whole-exome sequencing using the formalin-fixed paraffin-embedded (FFPE) tumor tissue DNA from 46 patients. TMB was then calculated, and patients divided equally into two groups based on the median TMB value: TMB-high group (TMB-H, > 4 mutations/Mb, n=23) and TMB-Low group (TMB-L, < 4 mutations/Mb, n=23). The TMB-H group showed a higher ratio of patients with a partial response (PR) (22% vs. 9%), while the TMB-L group showed a higher ratio of patients with progressive disease (PD) (78% vs. 26%) (**Figure 1A**). The PR and stable disease (SD) patients both exhibited significantly higher TMB than PD (**Figure 1B**; **Supplementary Table S1**). A significantly higher proportion of TMB-H patients displayed progression-free survival (PFS) than TMB-L patients. [*P* = 0.0003, hazard ratio (HR) = 3.76, 95% confidence interval (CI) 1.85 - 7.64] (**Figure 1C**). These results are in concordance with previously reported findings from other tumor types (15).

We next examined the influence of HLA-I heterogeneity on the therapy response in NPC patients using high-resolution HLA class I genotyping of blood DNA samples from the 46 patients. The diversity of HLA in NPC patients was accessed by examining the hetero-/homozygous alleles of HLA-A, HLA-B, and HLA-C. In this study, 38 of the NPC patients were classified as ‘Hetero’, as all three HLA-I loci were heterozygous, and eight patients as ‘Homo’ since at least one HLA-I locus was classified as homozygous. The HLA-I Homo group patients had a higher PD ratio than the HLA-I Hetero group (75% vs. 47%); the latter showing a higher PR and SD ratio (**Figure 1D**). A trend of reduced PFS was found for the HLA-I Homo group (*P* = 0.15, HR = 1.84, 95% CI 0.8 – 4.62) (**Figure 1E**). This statistical insignificance may likely be due to the limited HLA-I Homo sample size within this cohort. Patients with both heterozygous HLA-I and high TMB load presented improved PFS compared with patients that have homozygous HLA-I and low TMB load. (P = 0.0013, HR = 7.75, 95% CI 2.22-27.04) (**Figure 1F**).

HLA supertypes have also been reported to affect PFS in White populations by Chowell. et al. (16). Considering the different MHC haplotypes in different populations, we investigated the effect of the dominant HLA supertypes in our cohort. However, we found no significant associations between HLA supertypes and the clinical response (**Supplementary Figures S1F–I**), which is likely due to the limited sample size in this study. Nevertheless, we did observe a distribution of the HLA supertypes that differed from that previously reported in Whites. Both the 46 NPC patients and 500 healthy Chinese from another study by Zhou. et al. (27) have demonstrated higher ratio of HLA-B62 supertype and lower ratio of HLA-A01 supertype, comparing with White population studied by Chowell. et al. (**Supplementary Figures S1C–E**).

### Association between plasma EBV DNA load and treatment response

**Figure 2:**
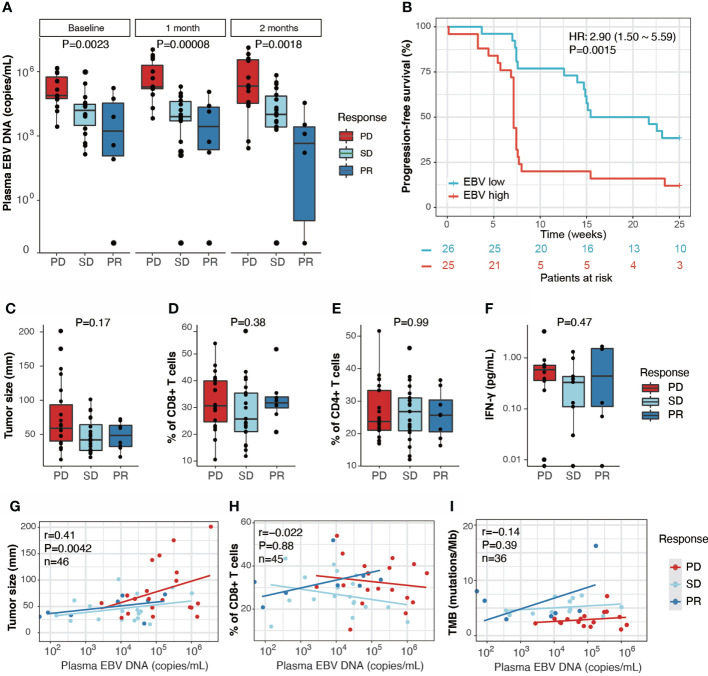
Plasma EBV DNA load is associated with response to anti-PD1 immunotherapy in RM-NPC patients. **(A)** Distribution of plasma EBV DNA copy number quantified by real-time polymerase chain reaction (qPCR) in PR (n=7), SD (n=19) and PD (n=25) patients. Plasma EBV DNA was assessed before treatment (Baseline) and post-treatment (1 month, 2 months). *P* values were calculated by using the Kruskal-Wallis test. **(B)**. Progression-free survival analysis of patients with high plasma EBV DNA (>50,000 copies/mL, EBV high, n=25) and low plasma EBV DNA (<50,000 copies/mL, EBV low, n=26) at baseline. **(C–F)** Boxplots showing the distribution of tumor size **(C)**, % blood CD8+ T cells amongst peripheral mononuclear cells **(D)**, % blood CD4+ T cells amongst peripheral mononuclear cells **(E)**, and blood IFN-γ **(F)** in the PD, SD and PR patients before treatment. *P* values were calculated by using the Kruskal-Wallis test. **(G–I)** Scatter plots of Pearson’s correlation coefficients between plasma EBV DNA and tumor size **(G)**, blood CD8+ T cells **(H)** and TMB **(I)**, PD, SD and PR patients were assessed separately.

We evaluated the plasma EBV DNA load of the PR, SD, and PD patients before and after they received anti-PD-1 therapy. There are 51 samples have EBV test results in baseline, during which only one sample have undetectable EBV level. We observed that plasma EBV DNA load differed significantly between PD, SD, and PR groups, both before treatment (baseline) and at one month and two months post-treatment (**Figure 2A**). The difference between the three response groups was enlarged after therapy compared with the baseline. EBV level was increased in PD group after one month, but stable in SD and PR group. EBV level decreased in PR group after 2 months post treatment (**Figure 2A**). The 51 patients with baseline data were then divided into two groups based on the median value of their plasma EBV DNA load (EBV-high > 50,000 copies/mL, n=25 and EBV-low < 50,000 copies/mL, n=26). The EBV-high patients had reduced PFS (*P* = 0.0015, HR = 2.9, 95% CI 1.5 - 5.59) (**Figure 2B**). This result was consistent with previous findings (34).

We also examined the baseline tumor size, blood CD8+ and CD4+ T cells proportions, and plasma IFN-γ levels in different response groups and did not observe a significant difference (**Figures 2C–F**; **Supplementary Table S2**). However, we found that plasma EBV DNA load was weakly related to tumor size (n=46, r=0.41, *P*= 0.0042) (**Figure 2G**), which was consistent with a previous study (35). However, we did not detect any correlations between plasma EBV DNA load and blood CD8+ T cell proportion, CD4+ T cell proportion, or TMB, using Spearman coefficient analysis (**Figures 2H, I**).

### Certain baseline gut bacteria are more prevalent among patients with disease progression during anti-PD-1 treatment

To investigate the changes in the gut microbiota in the course of anti-PD-1 therapy in NPC patients, we performed shotgun metagenomics sequencing on fecal material collected in 50 of the patients at baseline and at several time points along the course of anti-PD-1 treatment (**Supplementary Figure S2**). For nine of the patients, antibiotic usage during the immunotherapy was recorded. Similar to a previous study (19), the nine patients receiving antibiotics had reduced PFS as compared to those not receiving antibiotics (**Supplementary Figure S3C**). We therefore examined the association between treatment response and the gut microbiota at baseline and during therapy in the 41 patients that did not receive antibiotics.

**Figure 3:**
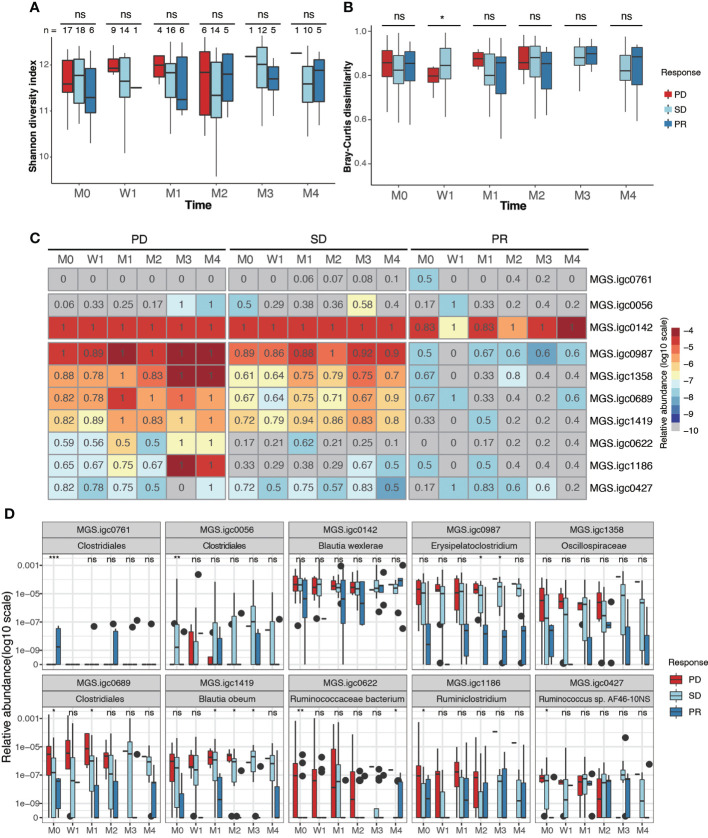
Identification of gut bacterial species associated with clinical response to anti-PD-1 therapy. **(A, B)**. Box plots showing Shannon alpha diversity **(A)** and Bray-Curtis dissimilarity **(B)** based on MGSs in fecal samples collected from PD, SD and PR patients during different stages of anti-PD-1 therapy. **(C)** Relative abundance (color) and prevalence (numerals) of differentially abundant MGSs among PR, SD and PD groups before treatment, as defined by analysis of variance (ANOVA). The color represents the median value (log10) of relative abundance within the response group. The prevalence rate is indicated as a numeral within the box. **(D)** Box plots showing the relative abundance of MGSs that exhibit differential abundance between groups. *P* values were calculated using the Kruskal-Wallis test (ns, non-significant; *P < 0.05; **P < 0.01; ***P < 0.001). M0: pre-therapy, W1: one-week post-treatment, M1-M4: 1-4 months post-treatment.

A highly diverse gut microbiota has been associated with preferred beneficial clinical response in melanoma, NSCLC, RCC and HCC (19, 20, 36), but not in GI cancer (21). In our NPC cohort, we found no significant differences in the diversity measures defined as Shannon diversity and Bray-Curtis dissimilarity between the treatment response groups (**Figures 3A, B**; **Supplementary Figure S3A, B**). These results were consistent before and after therapy.

We next analyzed if any MGSs were differentially prevalent and/or abundant before anti-PD-1 treatment among the PD, SD, and PR groups. After removing MGSs with prevalence lower than 20%, 10 MGSs were observed to be differentially enriched at baseline in at least one of the three groups (**Figures 3C, D**; **Supplementary Figure S3, Table 3**). Among the seven MGSs that were differentially enriched in the PD group at baseline, four were annotated at species level: *Blautia wexlerae* (MGS.igc0142), *Blautia obeum* (MGS.igc1419), *Ruminococcaceae bacterium* (MGS.igc0622), *Ruminococcus* sp. AF46-10NS (MGS.igc0427), and two were genus level annotated as *Erysipelatoclostridium* (MGS.igc0987) and *Ruminiclostridium* (MGS.igc1186). The remaining MGS, MGS.igc1358, belongs to the *Oscillospiraceae* family.

We found that most of the MGSs enriched in the PD group also displayed high or median abundance in the SD group. MGS.igc0761, annotated as a member of the *Clostridiales* order, was the only MGS enriched in PR, but it showed low abundance, and was only detected in 50% of the PR samples at baseline (**Figures 3C, D**).

### Differential gut bacterial pathways indicate potential mechanisms driving clinical responses

**Figure 4:**
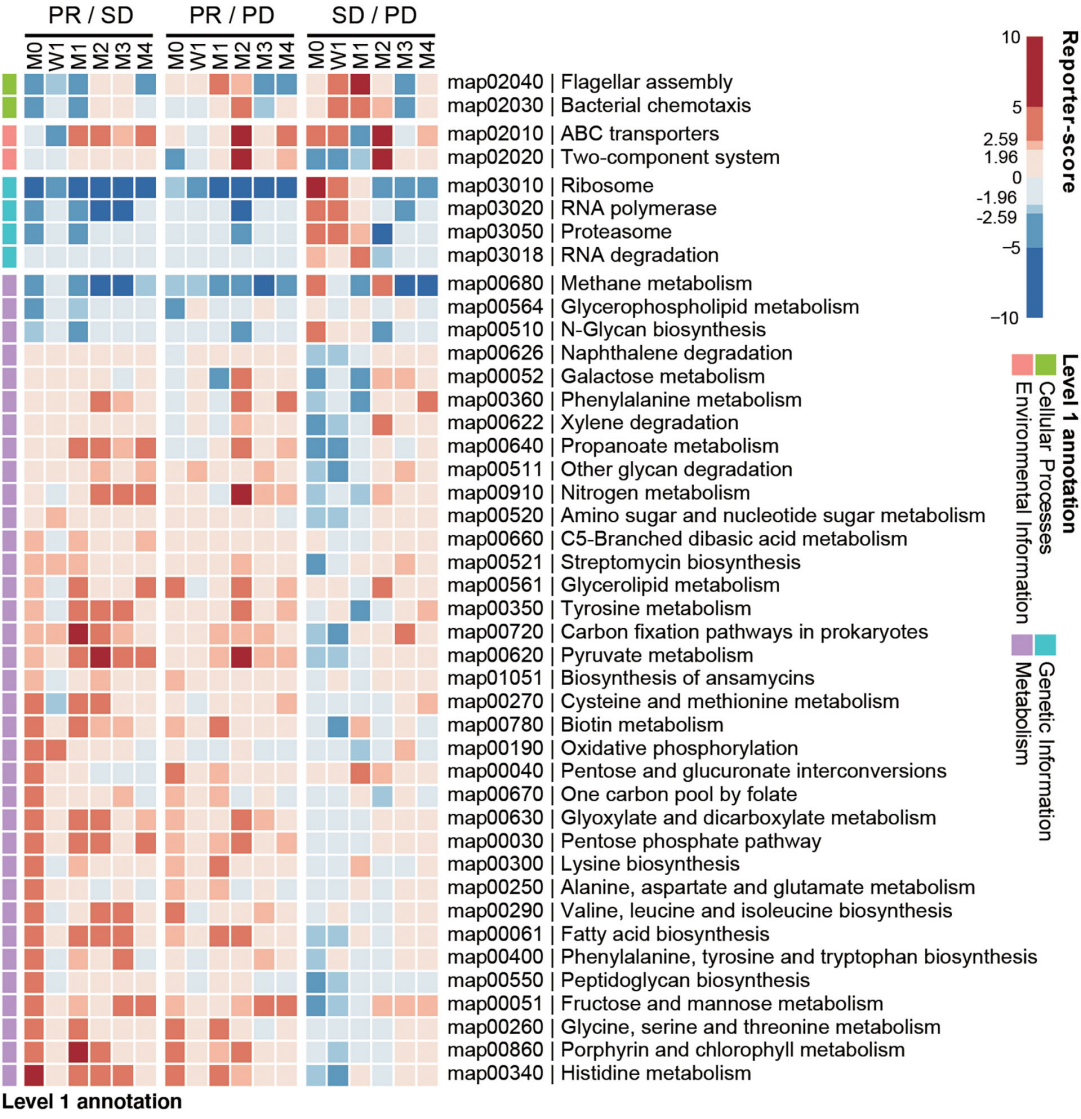
Baseline enrichment of gut microbial pathways in patients responding to anti-PD-1 therapy. KEGG-derived pathways from shotgun metagenomics data were computed by reporter-scores and compared pairwise between response groups. The enriched pathways were grouped based on the designated level 1 pathway categories. For each combination, a positive reporter-score (red) indicates the pathway was enriched in the response group (PR over SD and PD; SD over PD). A negative value (blue) indicates that the pathway was enriched in the non-response group. The significance of the differential enriched pathways was determined by reporter-score >1.96 or <-1.96 (equal to P < 0.05), and reporter score >2.59 or <-2.59 (equal to P < 0.01).

We next investigated if any gut microbial functions were differentially enriched among the PD, SD, and PR groups. We used a reporter-score pipeline (33) to quantify the difference of microbiome-related pathways based on KEGG orthologs (KO). We found that 32 biological functions related to bacterial metabolism were enriched in the PR group (**Figure 4**), including amino acid and fatty acid biosynthesis. Interestingly, these functions decreased in the PR group 1 week upon the first treatment. Functions related to genetic information, including ribosome, RNA polymerase, proteasome, and RNA degradation, were enriched in the PD and SD groups, but not in the PR group.

## Discussion

Anti-PD-1 immunotherapy has been reported to show prolonged benefit for metastatic cancer patients who have been treated with conventional therapy and acquired drug resistance. However, the main obstacle is that a substantial number of the patients does not respond to the anti-PD-1 therapy. The study of biomarkers related to efficient treatment responses is vital for patient screening and in furthering treatment efficacy. Gut bacteria have been shown to hold promises as biomarkers in relation to a number of cancer types (17–21), but so far, no studies on the potential role of the gut microbiota in relation to NPC patients have been reported. Here, we integrated multiple genetic and environmental factors, including TMB, HLA-I, blood EBV DNA load, blood T cells and IFN-γ, and longitudinal gut microbiome measures in an NPC cohort consisting of 57 patients.

In relation to genetic factors, we found that NPC patients with high TMB displayed improved clinical responses to anti-PD-1 therapy, as measured by PFS. A previous study by Wang. et al. (10) reported no correlations between TMB and PFS. The different results might be due to the different cohort settings, regimens, and methods used for the TMB analysis. Moreover, sequence artifacts from FFPE DNA have to be addressed when comparing FFPE DNA sequencing data from different studies, as the sequence artifacts could be induced by various tumor purity and DNA damage present in FFPE tissues (37). Except for TMB analysis, more variants analysis results about the WES data were reported in another study by us (38). In addition, we found HLA-I heterozygosity to positively contribute to PFS, which is in concordance to a previous report in melanoma patients (16). This result might be explained by the antigen presentation system of tumor cells, as HLA-I heterozygosity results in a higher diversity of MHC class I molecules on the cell surface, potentially presenting more different antigens that are recognized by cytotoxic T cells. The study of HLA in melanoma also reported specific HLA supertypes, including B44 and B62 to be correlated with prognosis. However, we did not identify specific HLA supertypes to influence the treatment response in our NPC cohort. Regardless, we found our NPC cohort and a healthy Chinese cohort to display a different pattern of HLA supertypes as compared to the White melanoma cohort. This result indicates that specific HLA supertype variations may benefit different populations. Additional and more extensive cohort studies in different populations are required to reveal if strong correlations exist between TMB and HLA supertypes and the prognosis for NPC patients. Besides that, tumor MHC expression needs to be taken into consideration in future studies. Tumor MHC expression plays significant roles in antigen presentation and it is reported to be used to guide immunotherapy selection in melanoma and NPC (39, 40).

Regarding environmental factors, we found that higher blood EBV DNA load was significantly related to poor clinical prognosis, as previously reported (41). Blood EBV load is often found to correlate positively with tumor size, reflecting that the cell-free EBV DNA likely originates from the tumor mass (35). Other than tumor size, the plasma EBV DNA load did not show correlation with the other identified markers (TMB, peripheral blood T helper cells and cytotoxic T cells, or IFN-γ levels).

The gut microbiome has been considered one of the most critical biomarkers in predicting the prognosis of anti-PD-1 therapy and has been widely studied in many tumors, but not yet in NPC. In this study, we characterized the gut microbiota in 50 RM-NPC patients that received anti-PD-1 treatment, and excluded 9 in the following analysis due to confounding effects from antibiotics. We found that responders and non-responders had no differences in gut microbiota diversity. Intriguingly, we identified several bacteria to be enriched in the non-responders, including *Blautia obeum*, and a bacterium belonging to the *Erysipelatoclostridium* genus, one to the *Oscillospiraceae* family, and one to the *Clostridiales* order, all with high abundance. The abundance of *Oscillospira* was previously reported to be significantly increased in NPC patients (42). However, these species need further experimental testing to validate their potential use as markers for non-responders in relation to cancer immunotherapy. To validate the differential enriched gut bacterium with different method, we performed a two-tiered random forest model using MGS profile from a training group of 16 samples (4 PD, 8 SD and 4 PR). Thirty-one feature MGSs were generated and we found five of them were previously identified (MGS.igc0056, igc0689, igc0622, igc1186 and igc0427) (**Supplementary Table S4**). Furthermore, we validate the 31 feature MGSs in a testing group of 22 samples (11 PD, 9 SD and 2 PR) and achieved great prediction performance with an AUC of 1 for PR, AUC of 0.96 for SD and AUC of 0.92 for PD (**Supplementary Figure S4**). Nevertheless, more samples especially responders of the anti-PD-1 therapy were required to increase the performance of prediction model in the future.

There are some limitations to our study. First, the sample size is small to precluding robust conclusions; especially the number of patients in the responder group is limited. Furthermore, the follow-up time of patients was short (25 weeks), which may affect the PFS analysis. Except for the factors included in the study, many other factors such as PD-L1 expression, immune-protein gene expression, microsatellite instability (MSI), and mutational pathways also need to be considered in order to provide comprehensive data of genetic and environmental markers. For lymphocytes analysis, tumor resident CD8+ T cells would be more relevant than blood CD8+ T cells. In spite of these limitations, we were able to establish a functional prediction model based on multiple parameters providing a platform for follow up studies on larger cohorts to benefit future NPC patients.

We conclude that both genetic and environmental factors may affect the anti-PD-1 therapy in NPC patients. In particular, TMB, HLA-I, blood EBV DNA load, and gut microbiota warrant examination when instigating ICI treatment to NPC patients.

## Data availability statement

The data presented in the study are deposited in the CNGB Sequence Archive (CNSA) of the China National GeneBank DataBase (CNGBdb) repository, accession number CNP0002119.

## Ethics statement

The studies involving human participants were reviewed and approved by Institutional review board on bioethics and biosafety of BGI NO. BGI-IRB 16051. The patients/participants provided their written informed consent to participate in this study.

## Author contributions

LZ, SB, KK, WF, LX, YM, and CF conceived the study. LX designed and implemented the study and prepared the manuscript. YM, and CF contributed to the implementation of the survey, data analysis and preparation of manuscript. ZP contributed to the data analysis and interpretation. FG contributed to the clinical information collection and interpretation. JM contributed to the metagenome sequencing data analysis. SQ, and QY contributed to the genetic data analysis. YH contributed to the design of the study. All authors contributed to the article and approved the submitted version.

## Funding

This study was supported by grants from: Shenzhen Municipal Government of China (No.KQJSCX20180329191008922), National Key R&D Program of China (2016YFC0905500 and 2016YFC0905503), Natural Science Foundation of Guangdong Province, China (No. 2018A030313379), Science and Technology Program of Guangdong (2017B020227001), Science and Technology Program of Guangzhou (201607020031).

## Acknowledgments

We would like to thank all the patients for their participation. We are grateful to all the participants who have made this research possible.

## Conflict of interest

The author’s LX and YH were employed by the company Latvia MGI Tech SIA.

The remaining authors declare that the research was conducted in the absence of any commercial or financial relationships that could be construed as a potential conflict of interest.

## Publisher’s note

All claims expressed in this article are solely those of the authors and do not necessarily represent those of their affiliated organizations, or those of the publisher, the editors and the reviewers. Any product that may be evaluated in this article, or claim that may be made by its manufacturer, is not guaranteed or endorsed by the publisher.
