# Supplementary material for: Genomic and microbial factors affect the prognosis of anti-pd-1 immunotherapy in nasopharyngeal carcinoma
Source: Front Oncol. 2022 Aug 17;12:953884. doi: 10.3389/fonc.2022.953884 (PMC9428750; doi:10.3389/fonc.2022.953884)
Supplement: Supplementary file 1 [file DataSheet_1.pdf]

Supplementary figures

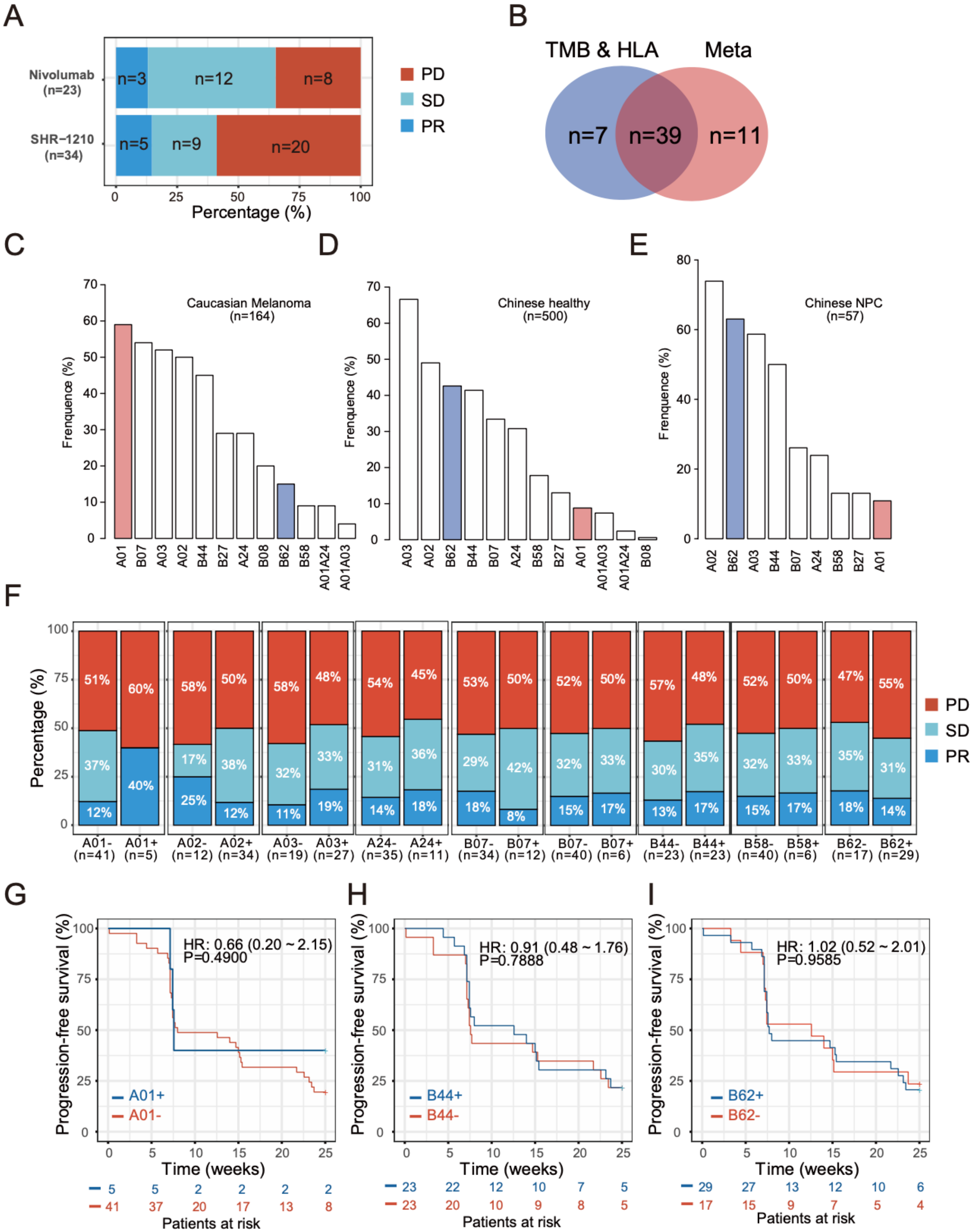

Supplementary figure 1. Patient cohort statistics and HLA supertype information. (A), clinical response of the patients that received anti-PD-1 therapy (Nivolumab or SHR-1210 (Camrelizumab)). (B), The relationship between fecal sample (Metagenome), tumor sample (TMB) and HLA data collection in the 57 patients. (C-E), Frequency of specific HLA supertypes in the referred White melanoma cohort (C), referred healthy Chinese cohort (D), and NPC cohort (E). (F), Clinical response rates within the NPC patients according to HLA supertypes. Plus (+) indicates the specific HLA supertype detected in at least one allele. Minus (-) indicates the HLA supertype negative for both alleles. (G-I), comparison of progression-free survival in NPC patients with and without A01, B44, and B62.

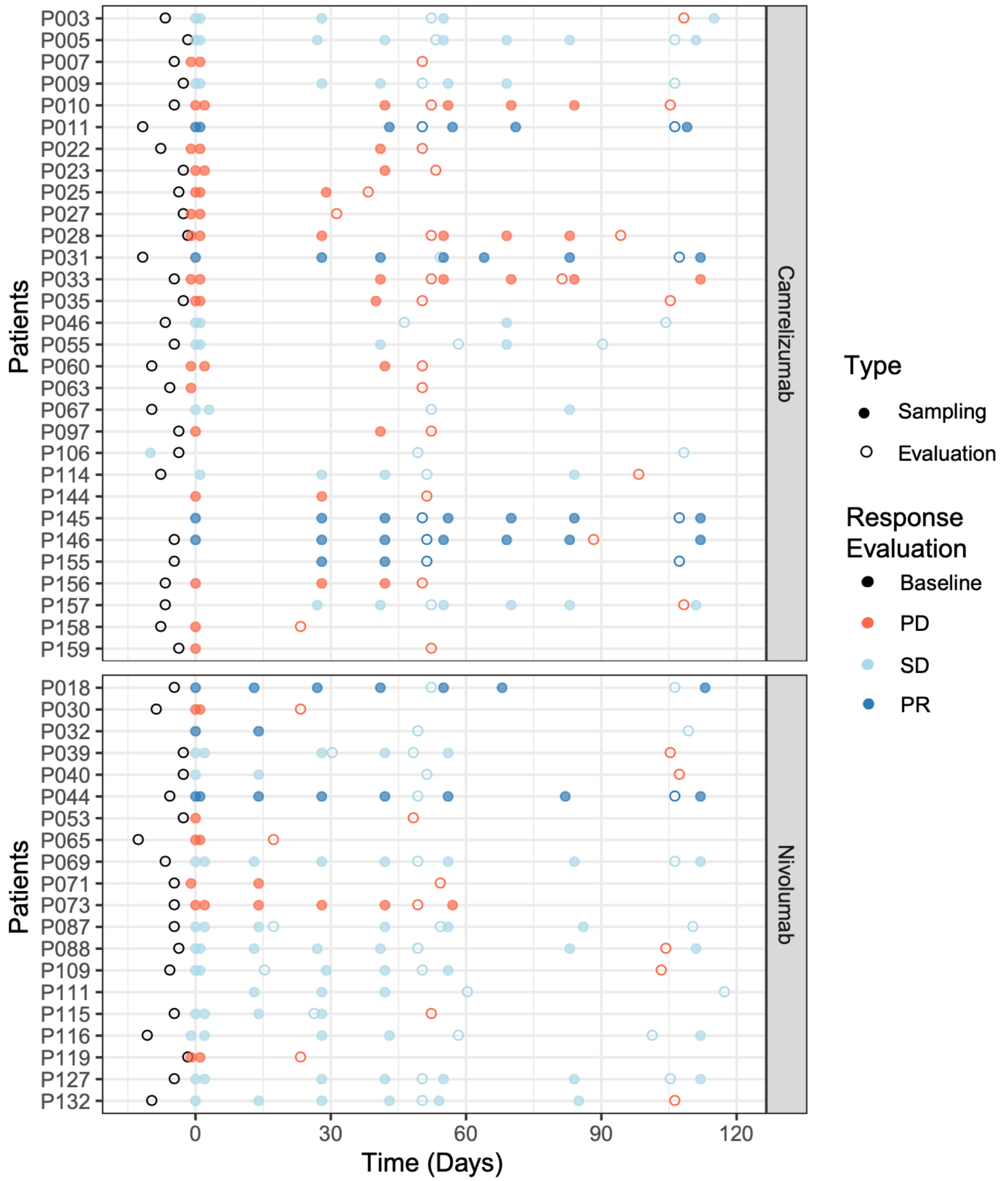

Supplementary figure 2. Overview of patient evaluations and fecal sample collection time points. Fecal samples were collected from thirty patients receiving SHR-1210 (Camrelizumab) and twenty patients receiving Nivolumab. Filled circles represent the sampling time point, and empty circles indicate the time of clinical evaluation. Evaluation results are showed with different colors (PD, Red circle; SD, light blue circle; PR, dark blue), and the black circle indicates the evaluation at baseline before therapy.

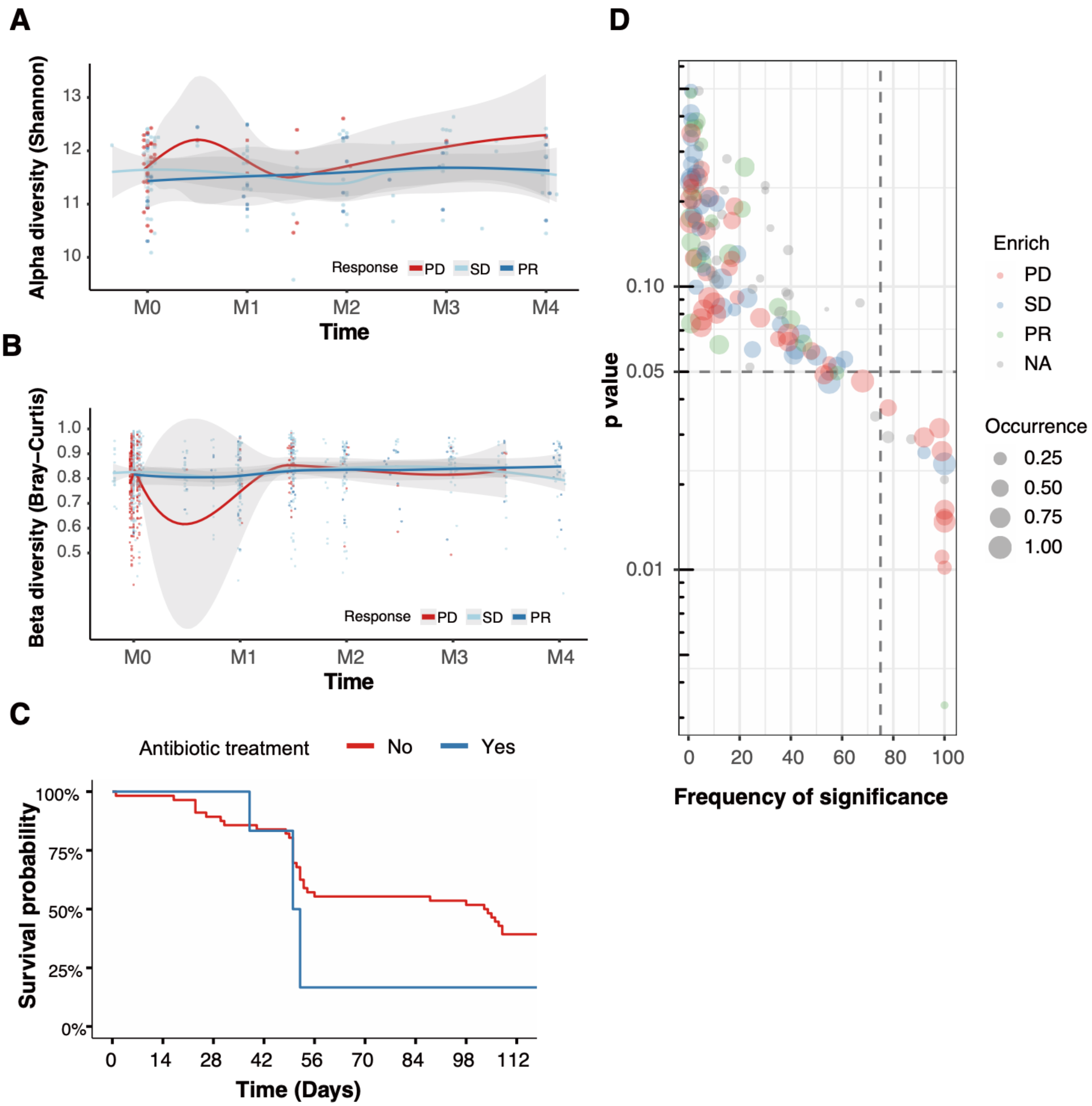

Supplementary figure 3. Features of the gut microbiota within the NPC patients. Dynamics of the alpha Shannon diversity (A) and Bray-Curtis beta diversity (B) of gut bacterial species (MGS) in PD, SD, and PR patients. M0, baseline; M1-M4, one to four months post-treatment. (C), survival probability of patients during the anti-PD-1 immunotherapy. Blue line represents the patients that received antibiotic treatment up to one month before the immunotherapy, and red line indicates the patients that did not receive antibiotic treatment. (D), statistics of the permutation test for MGSs within the PD, SD and PR groups at baseline. Threshold of *P* value was 0.05, threshold of significance frequency was 75%. Circle size indicates the occurrence of each MGS.

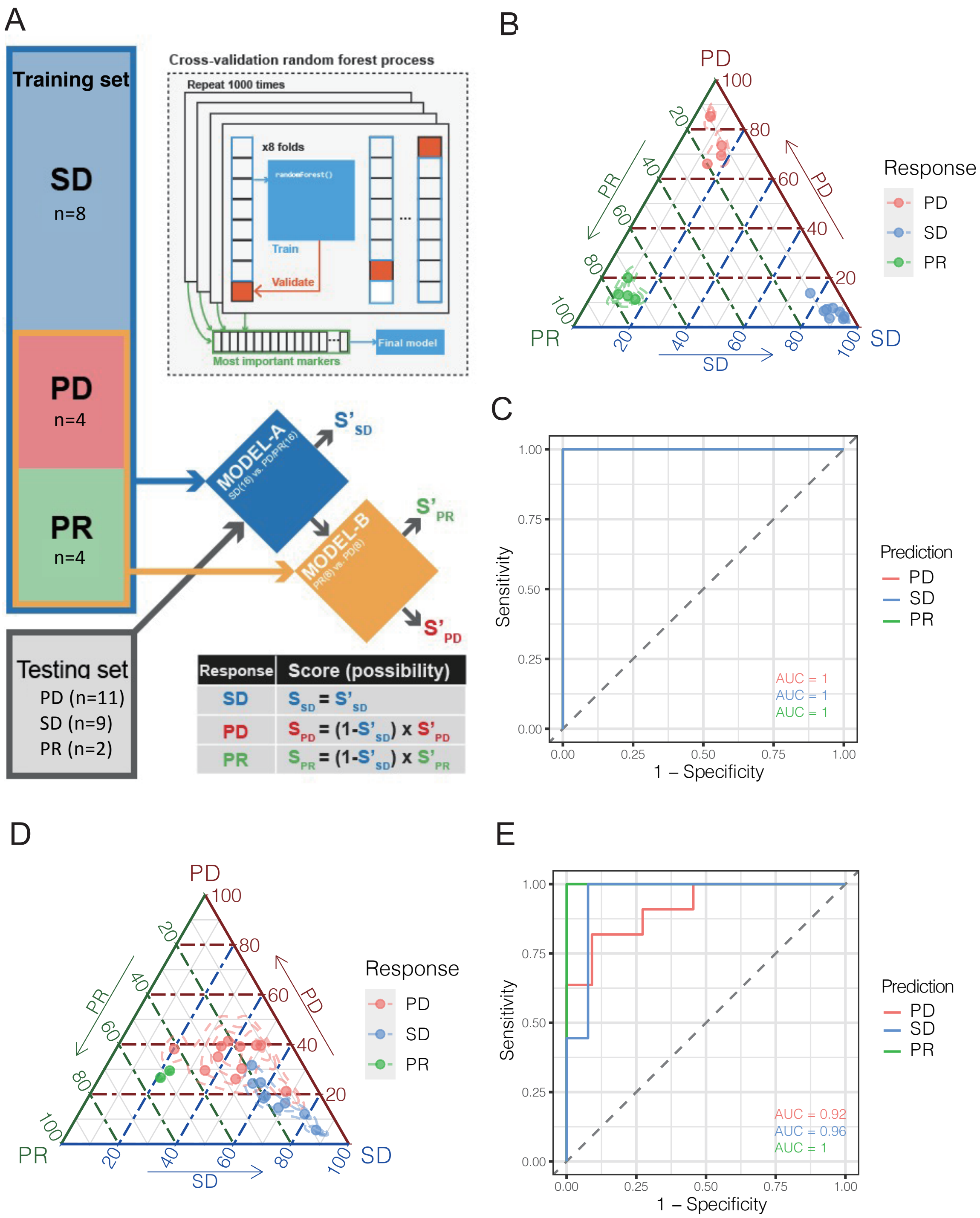

Supplementary figure 4. A random forest classifier for predicting anti-PD-1 response outcome using MGS profile. (A), A two-tiered random forest process with cross-validation for model training was implemented. The method includes two random forest models (Model-A and Model-B). Training set has 16 samples (4 PD, 8 SD and 4 PR), and 22 samples (11 PD, 9 SD and 2 PR) were used to test the model. (B, C), The classification of the response groups in the training group. All three response groups achieved an AUC as 1. (D, E), The classification of the response groups in the testing group, response prediction achieved AUC of 1 for PR, AUC of 0.96 for SD and AUC of 0.92 for PD.
